# Ventricular Fibrillation Arrest Triggered by Antiemetics Revealing an Underlying Long QT Syndrome in a Young Woman

**DOI:** 10.7759/cureus.64136

**Published:** 2024-07-09

**Authors:** Ameer Khan, Adel Kenawi, Sourabh Jadhav, Amsal Amjad, Muhammad Saleem

**Affiliations:** 1 Cardiology, Tameside General Hospital, Ashton-under-Lyne, GBR

**Keywords:** ecg interpretation, ventricular dysrhythmia, antiemetic agent, long qt, congenital long qt syndrome

## Abstract

Undiagnosed phenomena such as long QT syndrome can have devastating effects on patients. Our case, involving a woman in her 30s, highlights the serious effects of undiagnosed long QT and how antiemetic medications can precipitate cardiac events that can lead to fatalities. Various medications are known to prolong QT intervals, and clinicians must be aware of the side effects of some of these commonly used medications. While survival was achieved in this case, education and reflection can act as a tool to help improve global standards of care in this subgroup of the population.

## Introduction

Congenital long QT syndrome (LQTS) is a well-identified yet rare syndrome with proven associations to ventricular arrythmias leading to sudden cardiac death [1]. As a congenital cardiac syndrome, its prevalence is approximated at around one in 2500 [2]. The typical manifestations of the disease include a history of syncopal episodes accompanied by electrocardiograph evidence suggestive of prolonged QT intervals and T-wave abnormalities [2]. The QT interval is defined as the start of the Q wave to the end of the T wave on an electrocardiogram, representing the time taken for ventricular depolarization and repolarization. A prolonged QT value of over 440 ms is traditionally labeled as prolonged [2]. From an electrophysiology standpoint, the delayed cardiac repolarization phase in the cardiac cycle can lead to the development of fatal arrhythmias such as ventricular fibrillation and torsades de pointes [1].

Genetic advances in recent years have isolated specific genes responsible for the ion channels involved in the pathophysiology of the disease, with the KCNQ1 mutation associated with the most prevalent LQTS variant - LQTS 1 [1,2]. The genetic mutations can result in abnormal function of the ion channels and impact how ions, such as potassium, are transported across the cardiac cell membrane [2]. Potassium is an important ion involved in the repolarization of cardiac muscle, and gene mutations affecting these potassium channels can lead to a delayed cardiac repolarization phase in the cardiac cycle. This delay can subsequently lead to the development of fatal arrhythmias such as ventricular fibrillation and torsades de pointes [1].

However, as is the diagnostic conundrum faced by clinicians, the atypical presentations need meticulous monitoring, in-depth history, and genetic analysis to yield the answers to formulate an LQTS diagnosis [2]. In contrast to congenital LQTS, acquired LQTS can result from medications that interfere with the cardiac ion channels such as certain antiemetics [1,3]. Our case puts the spotlight on iatrogenic agents that can act as precipitants for life-threatening arrhythmias in LQTS patients.

## Case presentation

A 32-year-old female presented to the emergency department with multiple episodes of vomiting that had not been controlled by any means at home. This patient had numerous attendances to the hospital with similar symptoms and had been previously diagnosed with cyclical vomiting syndrome. Her presentation at this point included vomiting, nausea, and slight abdominal discomfort; however, there was no pyrexia, and inflammatory markers were within normal limits. She was seen by the acute medical team and diagnosed with viral gastroenteritis and was admitted for intravenous fluid resuscitation and medical optimization with appropriate antiemetics.

Her social circumstances involve being a single mother of three children with a background of anxiety. She was mobile and independent in her activities of daily living. During this admission, she had been given three different antiemetics including prochlorperazine 12.5 mg, ondansetron 4 mg, and cyclizine 50 mg to help control her vomiting.

She was moved to the acute medical unit overnight from the accident and emergency department. During the day shift, she experienced a witnessed tonic-clonic seizure, so an emergency call was made to help terminate the seizure. At this point, she became suddenly unresponsive. The crash team was called, and the advanced life support algorithm was commenced; she was found to be in ventricular fibrillation. Cardiopulmonary resuscitation was performed for one cycle, and the patient received a single shock to restore her to normal sinus rhythm. An arterial blood gas performed at the time showed a low potassium of 2.8 and a raised lactate of 9.7. She was then intubated and taken to the intensive care unit for further monitoring and treatment.

**Figure 1:**
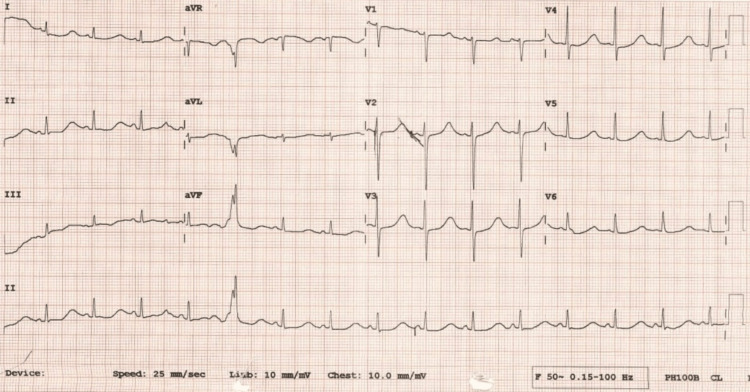
Electrocardiogram (ECG) showing a prolonged QTcB interval of 554 ms

A CT head scan was performed to check for any intracranial bleeding or abnormalities that could have precipitated the seizures, and it came back clear. Routine blood tests were performed to ensure no electrolyte abnormalities were present which could precipitate the arrest, and they came back normal. The patient spent five days in the intensive care unit before being stepped down to the acute coronary unit. On telemetry and review of her electrocardiograms (ECGs), a query was raised about LQTS (Figure 1). All proceeding ECGs post her cardiac arrest showed prolonged QT intervals, with the longest being recorded at 554 ms.

An echocardiogram was performed, which ruled out any valvular abnormalities and showed a preserved ejection fraction. Subsequently, a cardiac MRI scan was performed, which did not reveal any underlying ischemic or non-ischemic cardiomyopathies.

Electrophysiologists discussed the need for the implantation of an implantable cardiac defibrillator. Upon discharge, she was referred to the geneticist’s team to identify any underlying congenital abnormalities that precipitated this event. She was given cautionary advice about the suspicion of LQTS, and she received a list of medications that were known precipitants to prolong the QT interval. Upon follow-up of this case, it was found that the patient had genetic testing positive for congenital LQTS, leading to the implantation of an ICD (implantable cardioverter defibrillator).

## Discussion

LQTS can often masquerade as an asymptomatic condition, leaving patients unaware of the life-threatening risks it presents. Lifestyle modifications are essential in conjunction with medical and surgical interventions. In terms of medication, beta-blockers are the mainstay of pharmacological treatment, and they work by reducing the effect of the sympathetic nervous system on the heart, which results in a lower risk of arrhythmias [4]. For patients with a high risk of life-threatening arrhythmias, particularly those with a history of cardiac arrest, ICDs are recommended [4]. Therapies such as ICD implantation have helped to improve the outcomes of patients with LQTS. These implants can help avoid LQTS-associated tragedies. Numerous studies have shown the importance of such interventions, with one study showing that an ICD provided lifesaving shocks to more than 40% of the patient cohort with LQTS [5].

In this case of a young, active patient, it is important to consider the life-changing modifications necessary to ensure no such further episodes occur. Heightened awareness of the plethora of medications to avoid is essential to prevent drug-induced prolongations and, therefore, improve patient outcomes [6]. Clinicians must be aware of the different genotypes of LQTS as each genotype correlates with different arrhythmia triggers, which can be further explained to the patients [7]. In the genotype LQT1, patients are advised to avoid any strenuous exercise such as swimming or water sports [8]. A study by Schwartz et al. showed that exercise, particularly swimming, can be a trigger for arrhythmias in approximately 62% of the patients [8]. Furthermore, auditory stimuli were a major trigger for arrhythmias in those with the genotype LTQ2; hence, patients should be advised to avoid abrupt loud noises [8].

LQTS is influenced by multiple risk factors such as genetic mutations, patient demographics, and certain medical ailments. Genetically, a family history of sudden cardiac death, or LQTS, significantly increases the chance of developing LQTS; hence, genetic testing and cardiac evaluation are vital in relatives to ensure early detection and risk management. According to studies, the female gender is also a risk factor for the occurrence of severe arrhythmias in LQTS, which is thought to be associated with estrogen exposure through the menstrual cycle in post-pubertal women and estrogen replacement therapy in post-menopausal women [6,9]. However, studies thus far have been largely based on animal experiments. While preliminary research is being conducted, the clinical urgency to research the effects of gender disparity in congenital LQTS will help in risk stratification strategies in the future [9].

The mental health impact of LQTS also needs to be given full consideration. Studies have shown that LQTS patients had an increased prevalence of psychiatric comorbidities, including anxiety [10]. The weight of studies such as those conducted by Marstrand et al. adds to the importance of the holistic healthcare practice [10]. Increased risk of metabolic comorbidities, such as diabetes mellitus, further gives rise to other cardiovascular complications in this subgroup [10]. A multidisciplinary approach to the management of LQTS patients is vital for achieving the best outcomes in patients [11]. The involvement of genetic counselors, cardiologists, and psychiatrists can help provide the robust management plans needed to provide best-practice care for these patients [11].

The literature supports the notion that potential pharmacological triggers for prolonging the QT interval can have devastating consequences [12]. Orozco et al. highlighted the dangers of ondansetron usage and its role as a precipitant to life-threatening ventricular arrhythmias [12]. While ondansetron is generally considered safe and widely used for the treatment of nausea and vomiting, caution should be taken in patients with underlying cardiac disease or risk factors for long QT. An ECG can be carried out to help stratify the risk. Appropriate monitoring and appropriate dose selection are also important aspects to minimize the risk because higher doses of ondansetron are shown to increase the QT interval more than lower doses [3].

It is also important to discuss whether healthcare staff have adequate confidence in escalating concerns regarding a prolonged QT interval. A study by Abdalla and Khanra analyzing the ECG interpretation proficiency among medical doctors showed low levels of clinician confidence in interpreting ECGs [13]. This highlights a deficiency that needs urgent attention due to the importance of the investigation, especially since an abnormal ECG can lead to potentially life-threatening consequences. Continued education is paramount to ensure the safe management of patients with LQTS.

## Conclusions

With antiemetic prescriptions being a common practice, it is vital to educate about their side effects, such as prolongation of QT. Exercising future caution before using these medications will help mitigate the risks of such adverse events. While emergency departments are already so busy, routine ECGs could help prevent such disasters from happening. It is also essential to ensure that doctors are equipped with the necessary skills and experience for early recognition of such a phenomenon. A multilayered approach focusing on clinician education from undergraduate to postgraduate levels alongside multidisciplinary collaboration can help ensure the delivery of high-quality care going forward.
